# COVID-19 case fatality ratio and survival among hospitalized adults in Goiás, 2020: a cohort study

**DOI:** 10.1590/S2237-96222025v34e20240053.en

**Published:** 2025-05-23

**Authors:** Moara Alves Santa Bárbara Borges, Ana Laura de Sene Amâncio Zara, Lísia Gomes Martins de Moura Tomich, Adriana Oliveira Guilarde, Cacilda Pedrosa de Oliveira, Deborah Lopes Mota Carvajal, Marina Mascarenhas Roriz Pedrosa, Paulo Sérgio Sucasas da Costa, Marília Dalva Turchi

**Affiliations:** 1 Universidade Federal de Goiás, Instituto de Patologia Tropical e Saúde Pública, Goiânia, GO, Brazil; 2 Universidade Federal de Goiás, Hospital das Clínicas, Serviço de Infectologia, Goiânia, GO, Brazil; 3 Universidade Federal de Goiás, Faculdade de Farmácia, Programa de Pós-Graduação de Assistência de Avaliação em Saúde, Goiânia, GO, Brazil; 4 Sociedade Brasileira Israelita Albert Einstein, Hospital de Urgências de Goiás Dr. Valdemiro Cruz, Serviço de Infectologia, Goiânia, GO, Brazil; 5 Universidade Federal de Goiás, Faculdade de Medicina, Departamento de Clínica Médica, Goiânia, GO, Brazil; 6 Universidade Evangélica de Goiás, Faculdade de Medicina, Anápolis, GO, Brazil; 7 Hospital de Campanha para o Enfrentamento ao Coronavírus de Goiânia, Goiânia, GO, Brazil; 8 Hospital Estadual de Doenças Tropicais Dr. Anuar Auad, Goiânia, GO, Brazil; 9 Universidade Federal de Goiás, Faculdade de Medicina, Departamento de Pediatria, Goiânia, GO, Brazil

**Keywords:** Covid-19, Hospitalization, Mortality, Cohort Study, Brazil, COVID-19, Hospitalización, Mortalidad, Estudios de Cohorte, Brasil

## Abstract

**Objective:**

To describe clinical-epidemiological and therapeutic aspects and to estimate case fatality ratio and risk factors for lower in-hospital survival due to COVID-19.

**Methods:**

This is a retrospective cohort study conducted in the state of Goiás, Brazil, in 2020, with data obtained from the Influenza Epidemiological Surveillance Information System and through a review of clinical records and hospital. Relative risk for in-hospital death was estimated and Poisson multiple regression and Cox regression analyses were performed. Survival functions were compared using the log-rank test and represented by Kaplan-Meier curves.

**Results:**

The sample consisted of 651 adults, whose median age was 59 years, 57.0% were admitted to public hospitals, 61.1% had severe acute respiratory syndrome on admission and 72.0% had at least one comorbidity, the most frequent being hypertension , diabetes and obesity. The overall case fatality ratio was 17.5% (95% confidence interval, 95%CI 14.7; 20.6), with no significant difference between public and private hospitals. The case fatality ratio was higher in the ≥60 years age group (prevalence ratio, PR 1.26; 95%CI 1.01; 1.58), in hypertensive patients (PR 1.41; 95%CI 1.14; 1 .74) and in those undergoing intensive care (PR 2.68; 95%CI 1.13; 6.32) and mechanical ventilation (PR 11.15; 95%CI 5.53; 22.46). The median time between hospital admission and death was 10 days (interquartile range, 6-18). Survival was lower in the ≥60 years age group (adjusted hazard ratio, HR 1.93; 95%CI 1.26; 2.95) and in those undergoing mechanical ventilation (HR 10.13; 95%CI 6.03; 17. 02).

**Conclusion:**

Factors related to comorbidities and severity were independent predictors of shorter in-hospital survival among patients with COVID-19.

## Resumen

**Objetivo:**

Describir aspectos clínico-epidemiológicos y terapéuticos y estimar letalidad y factores de riesgo de menor supervivencia hospitalaria por COVID-19.

**Métodos:**

Se trata de un estudio de cohorte retrospectivo realizado en Goiás, Brasil, en 2020, con datos obtenidos del Sistema de Información de Vigilancia Epidemiológica de Influenza y mediante revisión de historias clínicas y hospitalarias. Se estimó el riesgo relativo de muerte hospitalaria y se realizaron análisis de regresión múltiple de Poisson y de regresión de Cox. Las funciones de supervivencia se compararon mediante la prueba de rangos logarítmicos y se representaron mediante curvas de Kaplan-Meier.

**Resultados:**

La muestra estuvo conformada por 651 adultos, cuya mediana de edad fue 59 años, el 57,0% ingresados ​​en un hospital público, el 61,1% con síndrome respiratorio agudo severo al ingreso y el 72,0% con alguna comorbilidad, siendo las más comunes hipertensión arterial, diabetes y obesidad. La letalidad general fue del 17,5% (intervalo de confianza del 95%, IC95% 14,7; 20,6), sin diferencias significativas entre hospitales públicos y privados. La letalidad fue mayor en el grupo de edad ≥60 años (razón de prevalencia, RP 1,26; IC95% 1,01; 1,58), en pacientes hipertensos (RP 1,41; IC95% 1,14; 1,74) y en cuidados intensivos (RP 2,68; 95%IC 1,13; 6,32) y ventilación mecánica (PR 11,15; IC95% 5,53; 22,46). La mediana de tiempo entre el ingreso hospitalario y la muerte fue de 10 días (rango intercuartílico, 6-18). La supervivencia fue menor en el grupo de ≥60 años (índice de riesgo ajustado - *hazard ratio*, HR 1,93; IC95% 1,26; 2,95) y en aquellos bajo ventilación mecánica (HR 10,13; IC95% 6,03; 17,02).

**Conclusión:**

Los factores relacionados con las comorbilidades y la gravedad fueron predictores independientes de menor supervivencia hospitalaria en pacientes con COVID-19.

**Ethical aspects**

This research respected ethical principles, having obtained the following approval data:

**Research Ethics Committees**: **Opinion Numbers**, **Approval Dates**

Hospital das Clínicas da Universidade Federal de Goiás: 4515457 5112519, 29/1/2021 18/11/2021

Hospital e Maternidade Dona Íris: 4869484 5301886, 27/7/2021 21/3/2022

Leide das Neves Ferreira (Secretaria Estadual de Saúde): 5171067, 16/12/2021

Informed Consent Form: Exempted by the Committees

## Introduction

COVID-19 is a serious systemic disease with multiple forms, variable symptoms and outcomes, differing according to the affected population, epidemiological scenarios, circulating variants and vaccination status (1). The COVID-19 pandemic has posed significant challenges to healthcare systems around the world. Brazil, one of the most affected countries, faced unique issues due to its large population, size and regional disparities in healthcare infrastructure, and socioeconomic vulnerability (1,2). In Brazil, the number of cases reached more than 37 million and around 703,000 deaths by June 2023 (3). 

The COVID-19 case fatality ratio in Brazil was estimated at 35% to 38% for hospitalized patients, reaching 59% for those admitted to intensive care units and up to 80% for those mechanically ventilated (4,5). Regional differences can be seen in case fatality ratios and in the clinical profile of individuals hospitalized in different phases of the pandemic (2,4-6). Although there is vast literature using national or regional data, to date, few studies have analyzed in detail the clinical aspects and therapeutic management of patients admitted to public and private hospitals in the Midwest region of Brazil (7).

This study aimed to describe clinical-epidemiological aspects and therapeutic management, in addition to estimating the case fatality ratio and risk factors for death and lower in-hospital survival in adults hospitalized due to COVID-19 in the state of Goiás, Brazil, in 2020. 

## Methods

### Study type

This is a retrospective cohort study. 

### Background

The study was carried out in three public hospitals and two private hospitals in Goiânia and Anápolis, in the state of Goiás, Brazil. These hospitals were part of the specialized care network for patients with COVID-19. In 2020, 34 health units had intensive care beds for adults with COVID-19, of which 27 were in Goiânia and 7 in Anápolis. Of these, 20 were public and 14 were private. The state of Goiás has an estimated population of 7 million inhabitants, with Goiânia being the capital (1,437,366 inhabitants) and Anápolis being the largest city outside the capital’s metropolitan region (398,869 inhabitants) (8). 

### Participants

The study included individuals aged ≥18 years, hospitalized between March and December 2020 due to COVID-19, confirmed by molecular testing or antigen testing. Patients who remained hospitalized for less than 24 hours or were transferred were excluded from the sample.

### Variables

The primary outcome was in-hospital death. The secondary outcomes were: admission to intensive care units, need for mechanical ventilation, vasoactive drugs, hemodialysis, length of stay until death in hospital or hospital discharge, ventilation duration, time in intensive care and case fatality ratios by subgroup.

The exposure variables were grouped as follows.

a) Sociodemographic data: sex (female, male); age group (in years: <30, 30-59, ≥60); race/skin color (White, mixed race/Black, other); hospital (public, private); and hospitalization period (March-May, June-August, September-December).
b) Underlying conditions (categorized dichotomously – yes, no): comorbidity; obesity; hypertension; diabetes; chronic obstructive pulmonary disease; chronic kidney disease; and pregnancy.
c) Symptoms (categorized dichotomously – yes, no): fever; dyspnea; and severe acute respiratory syndrome on hospital admission.
d) Chest imaging exam (radiography and computed tomography): pulmonary involvement (<50%, ≥50%), based on the tests performed.
e) Therapeutic (categorized dichotomously – yes, no): hospitalization in intensive care; need for mechanical ventilation; use of antibiotics; use of corticosteroids; use of vasoactive drugs; use of heparin; and need for hemodialysis. Use of full doses of heparin (1mg/kg, every 12 hours) was compared to use of prophylactic doses of heparin (1mg/kg/day), among those who used heparin.
f) Complementary tests (values ​​with greater changes during hospitalization): anemia (hemoglobin <12g/L, ≥12g/L); leukocytosis (leucocytes>10,000 cells/µL, 4.000-10,000 cells/µL); lymphopenia (lymphocytes <1,000 cells/µL, ≥1,000 cells/µL); thrombocytopenia (blood platelets <100,000 cells/µL, ≥100,000 cells/µL); liver enzyme changes (alanine aminotransferase and aspartate aminotransferase >40 U/L, ≤40 U/L); lactate dehydrogenase (>328 U/L, ≤328 U/L); kidney injury (creatinine ≥1.4mg/dL, <1.4mg/dL); and D-dimer (≥ 500 µg/L, <500 µg/L).

### Data source

The data were obtained from the Influenza Epidemiological Surveillance Information System (*Sistema de Informação de Vigilância Epidemiológica da Grip*) and through a review of clinical records and hospital records. Clinical characteristics, complementary tests and therapeutic management were assessed, from admission to hospital discharge or death in hospital. The cases were categorized according to the World Health Organization clinical classification (9) as mild, moderate, severe and critical. 

The study data was collected and managed using the electronic data capture platform (Research Electronic Data Capture – REDCap), hosted on the server of the Federal University of Goiás. The data form was created by the researchers, and data collection was carried out by professionals trained in the health sector. 

### Study size

We performed non-probability sampling of patients admitted to each of the participating hospitals. We estimated that a sample of 650 people would be sufficient to estimate a relative risk (RR) of 1.80 for the primary outcome (death during hospitalization), taking into account the presence of at least one comorbidity. The parameters used were a two-tailed 95% significance level (1-alpha); 80% power (1-beta); ratio between exposed and unexposed equal to 2; and 15% of those not exposed and 24% of those exposed presenting the primary outcome.

## Statistical methods

Data were analyzed using IBM SPSS 20.0 software. The median and interquartile range (IQR) were calculated for continuous variables and absolute (n) and relative frequencies (%) were described for categorical variables. The case fatality ratio was calculated by the number of hospitalized individuals who died during hospitalization in the different subgroups. In order to identify factors associated with death, initially, we calculated the RR, with their respective 95% confidence intervals (95%CI), for each variable. The RR data referring to the laboratory tests were presented separately.

The Poisson multiple regression model with robust variance estimator was used to analyze the predictive factors of fatality, presented with their prevalence ratios (PR) and 95%CI; p-value<0.05 was considered statistically significant for all tests. Collinear variables or variables with missing values greater than 10% ​​(laboratory tests, chest imaging and heparin) were not included in multiple analyses, nor were those with a p-value≥0.10 in the bivariate analysis.

The survival functions of the groups were compared using the log-rank test and represented graphically by Kaplan-Meier curves per age group (in years: <60, ≥60), hypertension, kidney injury (creatinine ≥1.4), mechanical ventilation, intensive care and pregnancy variables. This analysis was complemented using the bivariate Cox regression model, obtaining the crude hazard ratio (HR). Variables with p-value≤0.10 in the bivariate analysis were included in the Cox proportional hazards model to adjust for possible confounding factors. Cox proportional hazards analysis was performed in a backwards model, including the sex, age, comorbidities, severe acute respiratory syndrome on admission, need for intensive care and mechanical ventilation variables. The magnitude of the final effect was obtained by presenting the HR and the 95%CI. Level of significance was obtained using the Wald test (p-value<0.05). The Schoenfeld test was used for residue analysis.

## Results

The study included 651 adults with COVID-19: 50.5% were female, 63.6% were hospitalized from June to August 2020 and 57.0% were admitted to a public hospital. Median age was 59 years (IQR 44-71 years). Of the 651 adults with COVID-19, 61.1% were admitted with severe acute respiratory syndrome, 56.0% were classified as severe or critical according to the World Health Organization and 72.0% had at least one comorbidity, the most common morbidities being hypertension, diabetes and obesity (Table 1). 

**Table 1:** Case fatality ratio (%) by subgroup, relative risk (RR) and 95% confidence intervals (95%CI) for the study population sociodemographic characteristics and clinical conditions. Goiás, 2020 (n=651)

Characteristics	Total n (%)	Case fatality ratio n (%)	RR (95%CI)	p-value^a^
Sex				
Female	329 (50.5)	53 (18.9)	1.00	0.342
Male	322 (49.5)	61 (16.1)	1.03 (0.96; 1.11)	
**Age group** (years)				
<30	47 (7.2)	2 (4.3)	1.00	<0.001
30-59	291 (44.7)	29 (10.0)	1.06 (0.99; 1.14)	
≥60	313 (48.1)	83 (26.5)	1.30 (1.19; 1.42)	
**Race/skin color**				
White	104 (37.0)	17 (16.3)	1.00	
Mixed race/black	162 (57.7)	27 (16.6)	0.99 (0.89; 1.11)	0.945
Other	15 (5.3)	-		
Hospital				
Private	280 (43.0)	48 (17.1)	0.96 (0.68; 1.35)	0.830
Public	371 (57.0)	66 (17.8)	1.00	
**Hospitalization period**				
March-May	104 (16.0)	22 (21.2)	1.12 (0.99; 1.25)	
June-August	414 (63.6)	76 (18.4)	1.08 (0.99; 1.16)	0.058
September-December	133 (20.4)	16 (12.0)	1.00	
**Underlying conditions**				
Comorbidity (at least one)	469 (72.0)	100 (21.3)	1.17 (1.10; 1.25)	<0.001
Comorbidity absent	182 (28.0)	14 (7.7)	1.00	
Obesity present	163 (25.0)	30 (26.3)	1.01 (0.93; 1.10)	0.729
Obesity absent	488 (75.0)	84 17.2)	1.00	
Hypertension present	319 (49.0)	79 (24.8)	1.19 (1.10; 1.28)	<0.001
Hypertension absent	332 (51.0)	35 (10.5)	1.00	
Diabetes present	184 (28.3)	53 (28.8)	1.22 (1.11; 1.35)	<0.001
Diabetes absent	467 (71.7)	61 (13.0)	1.00	
Chronic obstructive pulmonary disease present	35 (5.4)	11 (31.4)	1.21 (0.97; 1.52)	0.030
Chronic obstructive pulmonary disease absent	616 (94.6)	103 (16.7)	1.00	
Chronic kidney disease (prior) present	31 (4.8)	11 (35.5)	1.29 (0.99; 1.68)	0.007
Chronic kidney disease (prior) absent	620 (95.2)	103 (16.6)	1.00	
Pregnancy present	36 (10.9)	4 (11.1)	0.94 (0.83; 1.06)	0.387
Pregnancy absent	293 (89.1)	49 (16.7)	1.00	
Symptoms				
Fever present	244 (37.5)	61 (25.0)	1.16 (1.07; 1.26)	<0.001
Fever absent	407 (62.5)	53 (13.0)	1.00	
Dyspnea present	539 (82.8)	107 (19.9)	1.17 (1.09; 1.25)	0.001
Dyspnea absent	112 (17.2)	7 (6.3)	1.00	
Severe acute respiratory syndrome on admission present	398 (61.1)	91 (22.9)	1.18 (1.10; 1.16)	<0.001
Severe acute respiratory syndrome on admission absent	253 (38.9)	23 (9.1)	1.00	
**Chest imaging exam**				
Lung involvement ≥50%	197 (33.9)	77 (39.1)	1.54 (1.37; 1.73)	<0.001
Lung involvement <50%	384 (66.1)	24 (6.2)	1.00	
**Therapeutic measures**				
Intensive care present	207 (31.8)	103 (49.8)	1.94 (1.69; 2.22)	<0.001
Intensive care absent	444 (68.2)	11 (24.7)	1.00	
Invasive mechanical ventilation present	107 (16.4)	96 (89.7)	9.40 (5.37; 16.46)	<0.001
Invasive mechanical ventilation absent	544 (83.6)	18 (3.3)	1.00	
Antibiotic present	579 (88.9)	110 (19.0)	1.17 (1.09; 1.25)	0.008
Antibiotic absent	72 (11.1)	4 (5.5)	1.00	
Corticosteroid present	445 (68.4)	87 (19.6)	1.08 (1.01; 1.16)	0.044
Corticosteroid absent	206 (31.6)	27 (13.1)	1.00	
Vasoactive drug present	96 (14.7)	76 (79.2)	4.47 (3.03; 6.61)	<0.001
Vasoactive drug absent	555 (85.3)	38 (6.8)	1.00	
Heparin present	539 (82.8)	97 (18.0)	1.03 (0.95; 1.13)	0.475
Heparin absent	112 (17.2)	17 (15.0)	1.00	
Full dose of heparin (1mg/kg every 12 hours)	61 (11.3)	23 (37.7)	1.36 (1.11; 1.65)	<0.001
Prophylactic dose of heparin (1mg/kg/day)	478 (88.6)	74 (15.4)	1.00	
Hemodialysis present	54 (8.3)	42 (77.8)	3.96 (2.40; 6.52)	<0.001
Hemodialysis absent	597 (91.7)	72 (12.0)	1.00	

^a^Chi-square test (p<0.05).

The case fatality ratio was 17.5% (95%CI 14.7; 20.6), with no statistically significant differences between public and private hospitals (p-value>0.05). There was a higher proportion of cases classified as serious or critical in public hospitals (69.0% versus 40.0%, p-value<0.001). Among patients who died, 88.0% had at least one comorbidity and the case fatality ratio was higher among those who had comorbidities compared to those who did not (21.3% vs. 7.7%; p-value<0.001). The case fatality ratio among patients in intensive care was 49.8% (RR 1.94, 95%CI 1.69; 2.22) while among those receiving mechanical ventilation it was 89.7% (RR 9.40, 95%CI 5.37; 16.46) (Table 1).

Pregnant women accounted for 5.5% (n=36), 35 of whom were admitted to public hospitals. Their median age was 24 years (IQR 24-) and median gestational age was 31 weeks (IQR 26-); all of them had at least one comorbidity. Admission to intensive care occurred among 36.1% of them, and mechanical ventilation among 22.2%. Their case fatality ratio was 11.1% (95%CI 3.10; 26.10).

Hemoglobin <12g/L; leukocytes >10,000 cells/µL; blood platelets <100.00/µL; as well as lactate dehydrogenase, aminotransferase, creatinine and D-dimer values ​​above reference values, showed association with higher risk of death in the bivariate analysis (Table 2).

**Table 2:** Case fatality ratio (%), relative risk (RR) and 95% confidence intervals (95%CI) for in-hospital deaths, per laboratory variables recorded during hospitalization. Goiás, 2020

Laboratory tests	Total n (%)	Deaths n (%)	RR (95%CI)	p-value^a^
Hemoglobin <12g/L	180 (51.9)	60 (33.3)	1.40 (1.25; 1.56)	<0.001
Hemoglobin ≥12g/L	167 (48.1)	11 (6.5)	1.00	
Leucocytes >10,000 cells/µL	185 (61.5)	69 (37.3)	1.54 (1.37; 1.73)	<0.001
Leucocytes ≤10,000 cells/µL	116 (38.5)	4 (3.4)	1.00	
Lymphocytes <1,000 cells/µL	180 (52.6)	32 (17.8)	0.91 (0.81; 1.01)	0.090
Lymphocytes ≥1,000 cells/µL	162 (47.4)	41 (25.3)	1.00	
Blood platelets <100,000/µL	56 (16.1)	22 (39.2)	1.40 (1.13; 1.74)	<0.001
Blood platelets ≤100,000/µL	291 (83.9)	43 (14.7)	1.00	
Lactate dehydrogenase >328 U/L	167 (75.2)	48 (28.7)	1.30 (1.15;1.47)	0.001
Lactate dehydrogenase ≤328 U/L	55 (24.8)	4 (7.2)	1.00	
Aminotransferase, alanine >40 U/L	158 (63.2)	42 (26.6)	1.19 (1.06; 1.35)	0.008
Aminotransferase, alanine ≤40 U/L	92 (36.8)	11 (11.9)	1.00	
Aminotransferase, aspartate >40 U/L	174 (70.4)	50 (28.7)	1.35 (1.21; 1.49)	<0.001
Aminotransferase, aspartate ≤40 U/L	73 (29.6)	3 (4.1)	1.00	
Creatinine ≥1.4mg/dL (kidney injury)	115 (36.3)	71 (61.7)	2.56 (2.03; 3.23)	<0.001
Creatinine <1.4mg/dL	202 (63.7)	4 (19.8)	1.00	
D-dimer ≥500 µg/L	182 (67.2)	57 (31.3)	1.39 (1.25; 1.55)	<0.001
D-dimer <500 µg/L	89 (32.8)	4 (4.5)	1.00	

^a^Chi-square test (p<0.05).

In the Poisson regression analysis with a robust estimator, the demographic and clinical variables that were independently associated with death were age ≥60 years (PR 1.26; 95%CI 1.01; 1.58), hypertension (PR 1, 14; 95%CI 1.14; 1.74), admission to intensive care (PR 2.68; 95%CI 1.13; 6.32) and need for mechanical ventilation (PR 11.15; 95%CI 5.53; 22.46) (Table 3). The laboratory results that predicted death were leukocyte count >10,000 cells/µL (PR 1.16; 95%CI 1.02; 1.32) and creatinine ≥1.4mg/dL (PR 1.65; 95%CI 1.31; 2.08) (Supplementary Table 1).

**Table 3:** Prevalence ratios (PR) and 95% confidence intervals (95%CI) adjusted for in-hospital death due to COVID-19 following Poisson regression with robust variance estimator. Goiás, 2020 (n=651)

Characteristics	PR (95%CI)	p-value^a^
Male sex	0.96 (0.80; 1.16)	0.696
Female sex	1.00	
Age ≥60 years	1.26 (1.01; 1.58)	0.039
Age <60 years	1.00	
Hypertension present	1.41 (1.14; 1.74)	<0.001
Hypertension absent	1.00	
Intensive care present	2.68 (1.13; 6.32)	0.025
Intensive care absent	1.00	
Invasive mechanical ventilation present	11.15 (5.53; 22.46)	<0.001
Invasive mechanical ventilation absent	1.00	

^a^Wald test (p<0.05).

Median time between hospital admission and death in hospital was 10 days (IQR 6-18) – 70.0% occurred within 14 days. Among those admitted to intensive care units, median hospital stay was 9 days (IQR 6-14 days), with 6 days (IQR 3-11 days) in intensive care and 7 days (IQR 4-13 days) in mechanical ventilation. Hospitalization time was longer among non-survivors when compared to those who were discharged from hospital (10 vs. 6 days). Those who died received more antibiotics (96.5% vs. 87.3%) and corticosteroids (76.3% vs. 66.7%) and used full dose heparin (23.7% vs. 8.6%).

**Figure 1:**
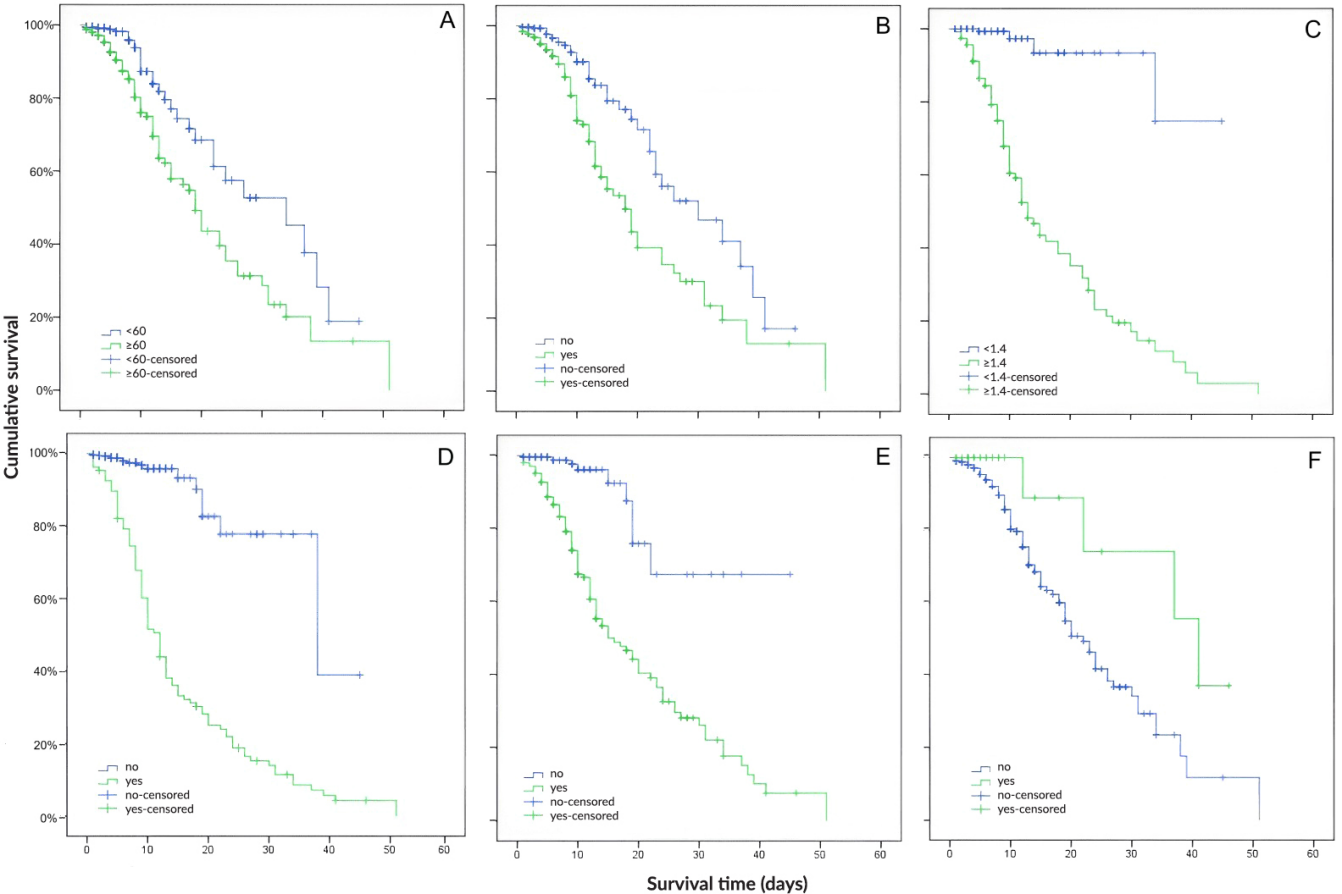
Survival time (hospitalization days) by death predictor variables – age (A), hypertension (B), kidney injury (C), mechanical ventilation (D), intensive care (E) e pregnancy (F) – during the hospitalization period. Goiás, 2020

**Figure 2:**
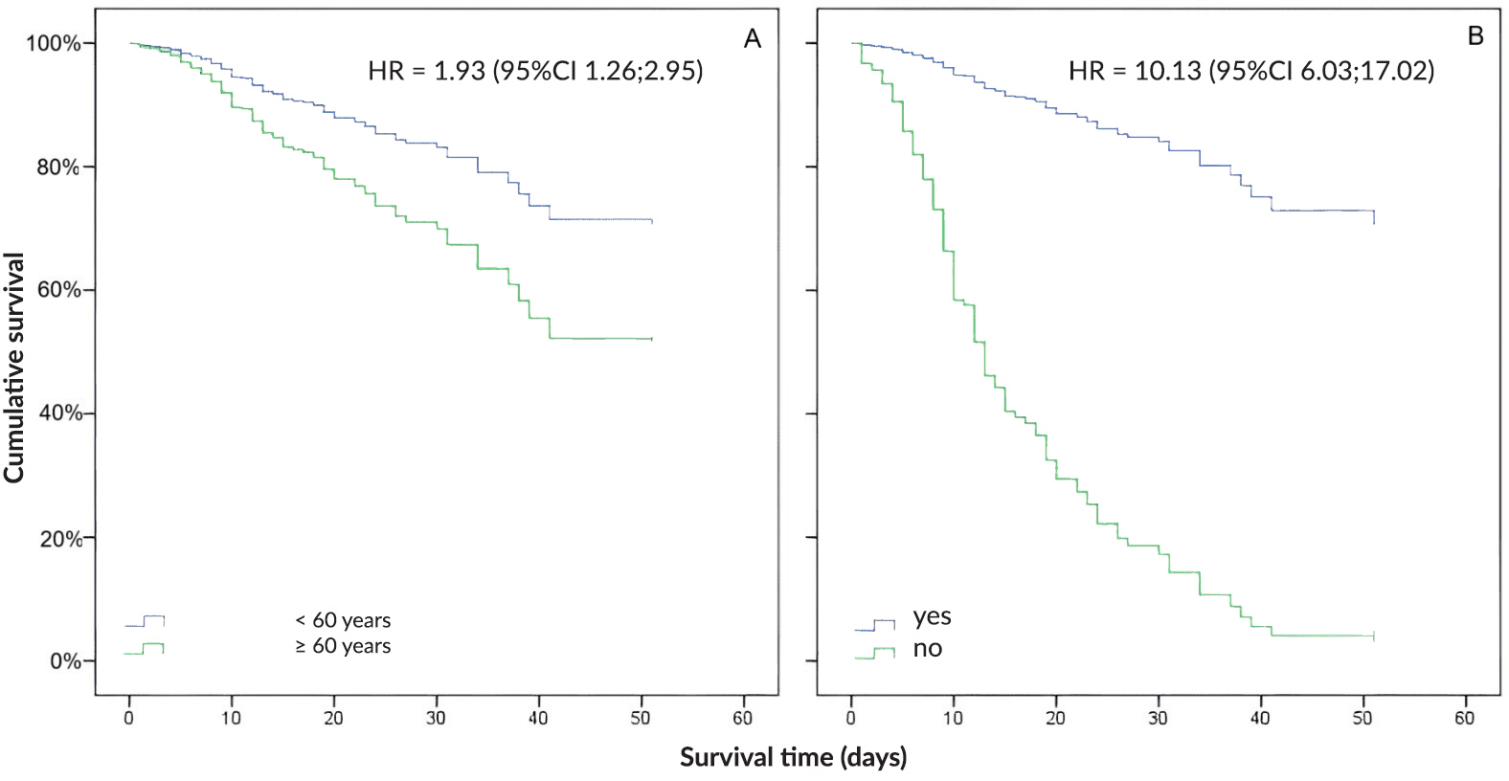
Survival according to age (A) and mechanical ventilation (B). Goiás, 2020 (n=651)

Age group, hypertension, kidney injury, pregnancy, need for mechanical ventilation and intensive care showed association with death in the bivariate Cox regression analysis (p-value<0.05) (Figure 1). The p-value and graphical representation of each variable obtained after the Schoenfeld test are available in the supplementary material (Supplementary Table 2 and Supplementary Figure 1). There was no violation of the proportional hazard assumption for the covariates used, except sex. After Cox proportional hazards analysis, only the age ≥60 years (HR 1.93; 95%CI 1.26; 2.95) and use of mechanical ventilation (HR 10.13; 95%CI 6.03; 17.02) variables were independently associated with lower in-hospital survival (Figure 2), even when only assessing patients who required intensive care.

## Discussion

In this study, we analyzed clinical, laboratory and therapeutic aspects and factors associated with the death of adults hospitalized due to COVID-19 in Goiás in the first year of the pandemic. Patients who died were older, had more comorbidities and presented greater clinical severity upon admission and need for intensive care. Age ≥60 years, hypertension and use of invasive ventilation were factors independently associated with higher case fatality ratios. Age and the presence of comorbidities are related to a greater need for advanced life care and unfavorable outcomes, including death, in people with COVID-19 (10,11). Adequate control of potentially modifiable factors, such as diabetes, hypertension, obesity, are essential for reducing the case fatality ratio due to severe respiratory syndromes. 

Similarities were found between the characteristics of this sample and studies conducted in the South and Southeast regions of Brazil in relation to the frequency of comorbidities (79.8%) and admissions to public hospitals (52.8%) (12,13). In those regions, the case fatality ratio ranged from 19.0% to 34.4% (5,12,13). In the North and Northeast regions of Brazil, the hospital case fatality ratio was 41.0% to 44.0%, reaching 96.0% in those undergoing mechanical ventilation in 2020 (5,14). There was a case fatality ratio of 21.0% (95%CI 18.0; 24.0) in hospitals in various regions of Brazil, ranging from 9.0% to 48.0%, with positive association between greater survival time and better economic indicators of the region where the patients were treated (15).

A multicenter cohort conducted in 25 Brazilian centers in 2020 found predictors for in-hospital case fatality ratio similar to the results of this research, such as age ≥65 years, hypertension, invasive ventilation and blood platelets <100,000 cells/L (13). In this study, laboratory changes such as leukocytosis, anemia, thrombocytopenia and elevation of transaminases, lactic dehydrogenase, D-dimer and creatinine represented risk of death (p-value<0.05).

No statistically significant differences were identified in the case fatality ratio between public and private hospitals, despite serious and critical presentations being more frequent in the public health network. Brazilian cohorts showed lower mortality among those admitted to private hospitals (13,16,17), but public hospitals received a higher proportion of high-risk or more severe patients (15).

During the first wave of the COVID-19 pandemic, the case fatality ratio reported in Brazil’s different regions ranged from 6.5% to 10.8% in private hospitals (13,16,17) and 21.7% in public hospitals, varying from 31.8% in the first wave to 18.2% in the second wave (18). This could be explained by regional disparities in terms of social inequalities and access to healthcare, coupled with periods of shortages of supplies, human resources and hospital beds faced by the healthcare system in the different phases of the pandemic (13,15,18).

In the COVID-19 pandemic, clinical management guidelines were established as evidence became available. In this study, 68.4% of patients used corticosteroids, while current guidelines recommend their use only in cases where oxygen is needed (9). Despite the low occurrence of effectively documented bacterial and fungal co-infections (19,20), 88.9% of patients used antibiotics, even higher than the 74.6% (95%CI 68.3; 80.0) reported in a meta-analysis (21). Excessive use of antimicrobial agents during the pandemic has already negatively impacted antimicrobial resistance rates and is considered to be a worrying legacy (22,23).

Although pregnancy was not identified as a risk factor for death in the multivariate analyses, the 11% case fatality ratio was of concern. As of June 2021, the overall COVID-19 case fatality rate among pregnant and postpartum women in Brazil was 7.2% (24). All pregnant women in this cohort could be considered high risk as they had at least one comorbidity. It should be noted that two of the study sites were referral services for pregnant women with COVID-19, which could have made them priority choices for severe cases and, thus, could have impacted the case fatality ratio in this population. 

This study had limitations inherent to retrospective studies, considering the quality and purpose of the available records. Using a convenience and non-probabilistic sample may not represent the population of hospitalized patients. Several risk factors were assessed dichotomously, without stratification by severity. In addition to this, there were differences in management between hospitals and incompleteness of data recorded on clinical records. In order to ensure uniformity, a standardized form was used to collect data, and medical records were reviewed by appropriately trained health professionals.

This study describes data from several hospitals in Goiás, with detailed clinical-epidemiological, laboratory and therapeutic information, including survival analysis. We concluded that factors related to comorbidities, such as advanced age and hypertension, as well as those associated with greater clinical severity, were predictors of death and shorter in-hospital survival. Understanding death risk factors and the regional disparities faced during the beginning of the COVID-19 pandemic in Brazil is essential for informing improvements in health management and providing input to reduce morbidity and mortality during the emergence of severe acute respiratory syndrome epidemics.

## Data Availability

The database used in this research is available at: https://figshare.com/s/6337256d2548a3f0ed5f.
