# Supplementary material for: COVID-19 case fatality ratio and survival among hospitalized adults in Goiás, 2020: a cohort study
Source: Epidemiol Serv Saude. 2025 May 23;34:e20240053. doi: 10.1590/S2237-96222025v34e20240053.en (PMC12105840; doi:10.1590/S2237-96222025v34e20240053.en)
Supplement: Supplementary file 1 [file 2237-9622-ress-34-e20240053-supp01-en.pdf]

Supplementary Table 1. Prevalence ratio (PR) and 95% confidence intervals (95%CI) for in-hospital death, by laboratory variables recorded during hospitalization, following Poisson regression with robust variance estimator. Goiás, 2020 (n=79)

Laboratory tests (n=79)	PR (95%CI)	p-value[a]
Hemoglobin <12g/L	1.12 (0.96; 1.32)	0.147
Hemoglobin ≥12g/L	1.00	
Leucocytes >10,000 cells/µL	1.16 (1.02; 1.32)	0.024
Leucocytes ≤10,000 cells/ µL	1.00	
Lymphocytes <1,000 cells/µL	0.89 (0.79; 1.01)	0.079
Lymphocytes ≥1,000 cells/µL	1.00	
Blood platelets <100,000/µL	0.91 (0.69; 1.19)	0.505
Blood platelets ≥100,000/µL	1.00	
Lactate dehydrogenase >328 U/L	1.05 (0.84; 1.31)	0.659
Lactate dehydrogenase ≤328 U/L	1.00	
Aminotransferase, alanine >40 U/L	1.02 (0.81; 1.29)	0.838
Aminotransferase, alanine ≤40 U/L	1.00	
Aminotransferase, aspartate >40 U/L	1.06 (0.84; 1.34)	0.590
Aminotransferase, aspartate ≤40 U/L	1.00	
Creatinine ≥1.4 mg/dL (kidney injury)	1.65 (1.31; 2.08)	<0.001
Creatinine <1.4 mg/dL (kidney injury)	1.00	
D-dimer ≥500 µg/L	1.11 (0.96; 1.28)	0.141
D-dimer <500 µg/L	1.00	

[a]Chi-square test (p<0.05).

Supplementary Table 2. P-values of covariates included in Cox proportional hazards analysis following Schoenfeld test

Variables	p-value
Sex	0.002
Age	0.791
Comorbidities	0.530
Mechanical ventilation	0.216
Intensive care	0.057
Severe acute respiratory syndrome	0.381

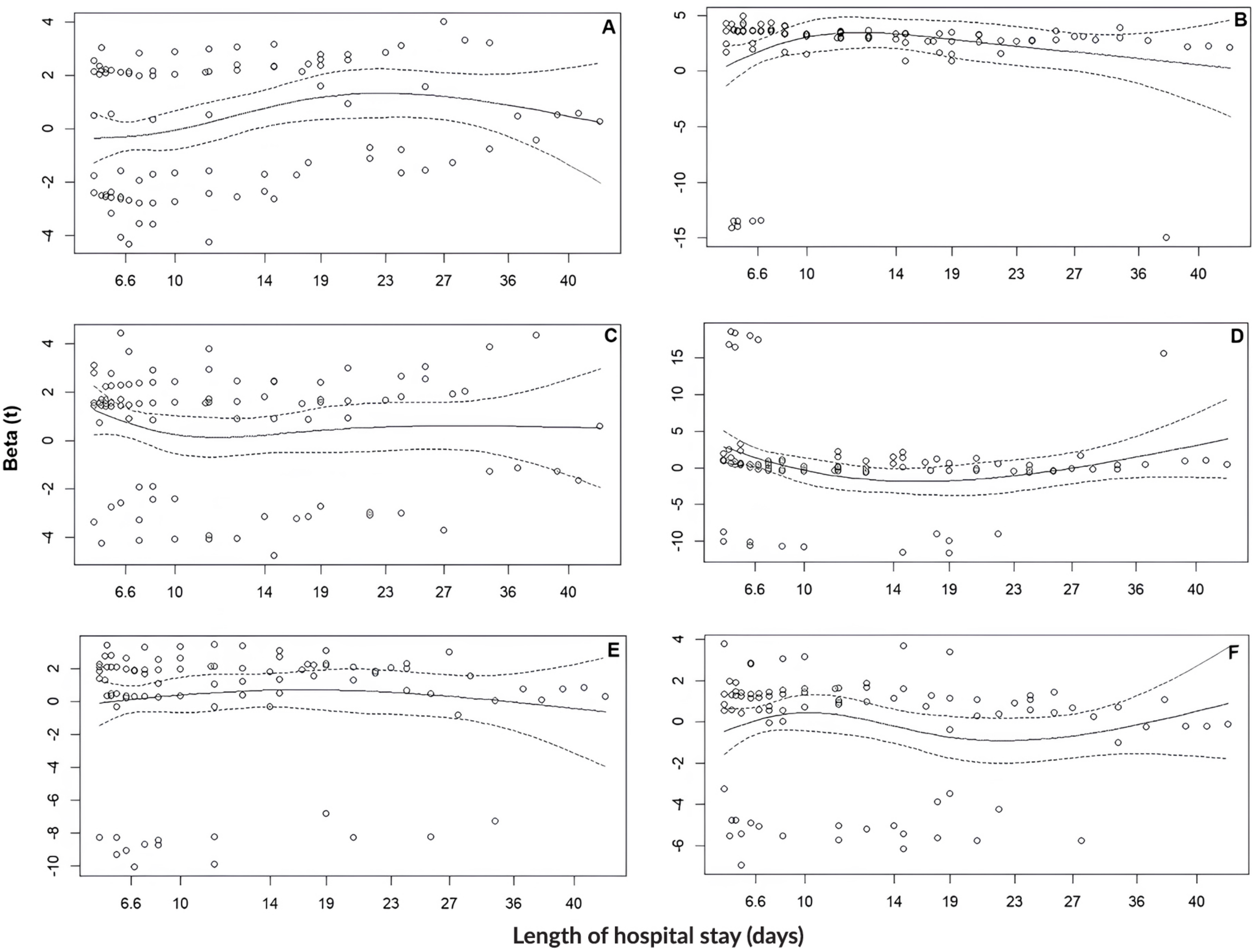

Supplementary Figure 1. Analysis of residues of the sex (A), mechanical ventilation (B), age < or ≥ 60 years (C), intensive care (D), comorbidities (E) and severe acute respiratory syndrome (F) covariates, according to the length of hospital stay for COVID-19 (months), used in the Cox proportional hazards analysis following the Schoenfeld test
