# Supplementary material for: COVID-19 case fatality ratio and survival among hospitalized adults in Goiás, 2020: a cohort study
Source: Epidemiol Serv Saude. 2025 May 23;34:e20240053. doi: 10.1590/S2237-96222025v34e20240053.en (PMC12105840; doi:10.1590/S2237-96222025v34e20240053.en)
Supplement: Supplementary file 2 [file 2237-9622-ress-34-e20240053-supp01-pt.pdf]

Tabela Suplementar 1. Razão de prevalência (RP) e intervalos de confiança de 95% (IC95%) para óbito intra-hospitalar, por variáveis laboratoriais registradas durante a internação, após regressão de Poisson com estimador de variância robusta. Goiás, 2020 (n=79)

Exames laboratoriais (n=79)	RP (IC95%)	p-valor[a]
Hemoglobina <12g/L	1,12 (0,96; 1,32)	0,147
Hemoglobina ≥12g/L	1,00	
Leucócitos >10.000 células/µL	1,16 (1,02; 1,32)	0,024
Leucócitos ≤10.000 células/µL	1,00	
Linfócitos <1.000 células/µL	0,89 (0,79; 1,01)	0,079
Linfócitos ≥1.000 células/µL	1,00	
Plaquetas <100.000/µL	0,91 (0,69; 1,19)	0,505
Plaquetas ≥100.000/µL	1,00	
Lactato desidrogenase >328 U/L	1,05 (0,84; 1,31)	0,659
Lactato desidrogenase ≤328 U/L	1,00	
Aminotransferase, alanina >40 U/L	1,02 (0,81; 1,29)	0,838
Aminotransferase, alanina ≤40 U/L	1,00	
Aminotransferase, aspartato >40 U/L	1,06 (0,84; 1,34)	0,590
Aminotransferase, aspartato ≤40 U/L	1,00	
Creatinina ≥1,4mg/dL (injúria renal)	1,65 (1,31; 2,08)	<0,001
Creatinina <1,4mg/dL (injúria renal)	1,00	
Dímero D ≥500 µg/L	1,11 (0,96; 1,28)	0,141
Dímero D <500 µg/L	1,00	

[a]Teste Qui-quadrado (p<0,05).

Tabela Suplementar 2. P-valores das covariáveis incluídas na análise de riscos proporcionais de Cox após teste de Schoenfeld

Variáveis	p-valor
Sexo	0,002
Idade	0,791
Comorbidades	0,530
Ventilação mecânica	0,216
Cuidados intensivos	0,057
Síndrome respiratória aguda grave	0,381

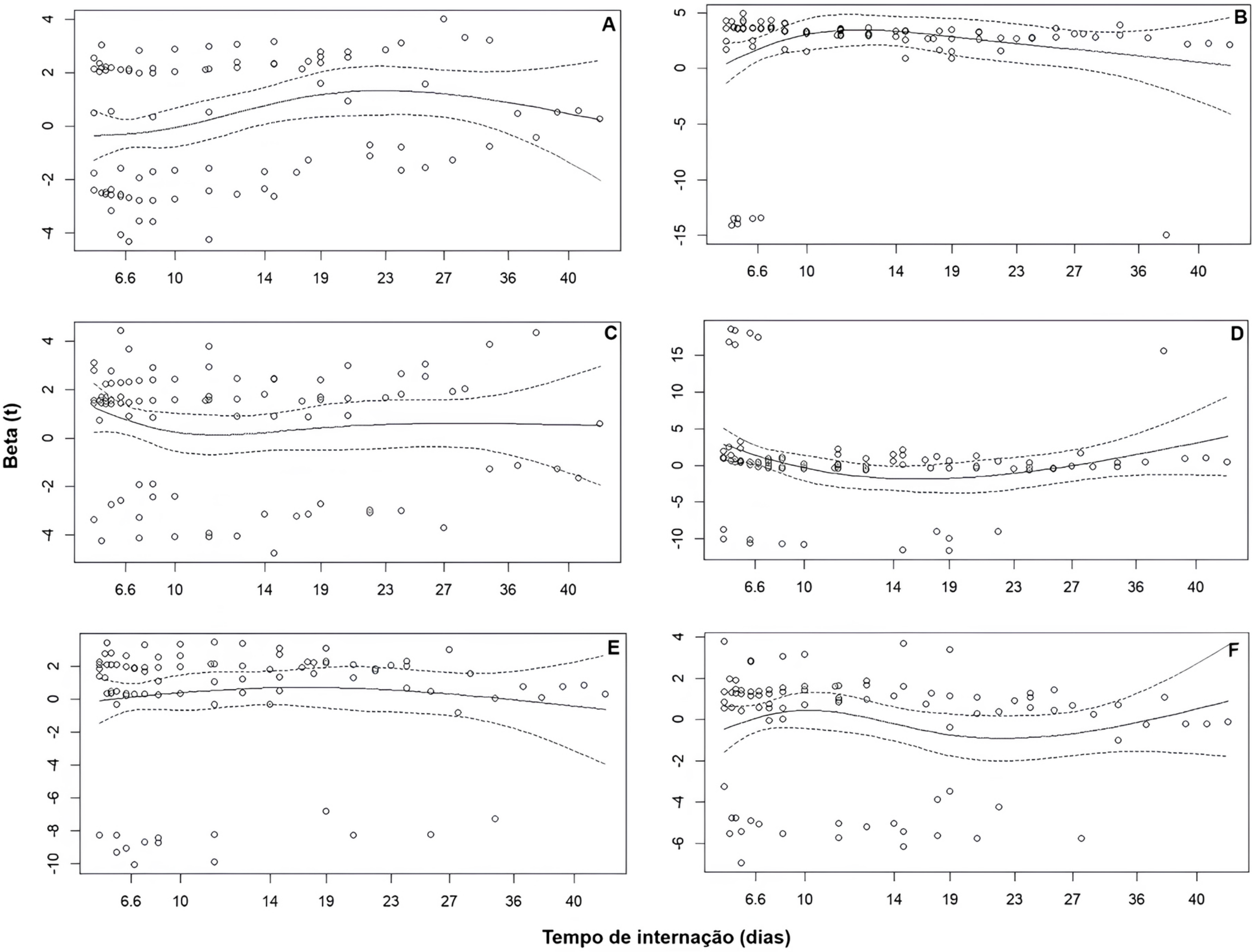

Figura Suplementar 1. Análise de resíduos das covariáveis sexo (A), ventilação mecânica (B), idade < ou ≥ 60 anos (C), cuidados intensivos (D), comorbidades (E) e síndrome respiratória aguda grave (F), de acordo com o tempo de internação por covid-19 (meses), utilizadas na análise de riscos proporcionais de Cox após teste de Schoenfeld
