# Supplementary material for: COVID-19 case fatality ratio and survival among hospitalized adults in Goiás, 2020: a cohort study
Source: Epidemiol Serv Saude. 2025 May 23;34:e20240053. doi: 10.1590/S2237-96222025v34e20240053.en (PMC12105840; doi:10.1590/S2237-96222025v34e20240053.en)
Supplement: Translation

# Translation: Letalidade e sobrevida por covid-19 em adultos hospitalizados em Goiás, 2020: um estudo de coorte

**DOI:** 10.1590/S2237-96222025v34e20240053.pt

**Authors:** Moara Alves Santa Bárbara Borges, Ana Laura de Sene Amâncio Zara, Lísia Gomes Martins de Moura Tomich, Adriana Oliveira Guilarde, Cacilda Pedrosa de Oliveira, Deborah Lopes Mota Carvajal, Marina Mascarenhas Roriz Pedrosa, Paulo Sérgio Sucasas da Costa, Marília Dalva Turchi

**Affiliations:** 1 Universidade Federal de Goiás, Instituto de Patologia Tropical e Saúde Pública, Goiânia, GO, Brasil; 2 Universidade Federal de Goiás, Hospital das Clínicas, Serviço de Infectologia, Goiânia, GO, Brasil; 3 Universidade Federal de Goiás, Faculdade de Farmácia, Programa de Pós-Graduação de Assistência de Avaliação em Saúde, Goiânia, GO, Brasil; 4 Sociedade Brasileira Israelita Albert Einstein, Hospital de Urgências de Goiás Dr. Valdemiro Cruz, Serviço de Infectologia, Goiânia, GO, Brasil; 5 Universidade Federal de Goiás, Faculdade de Medicina, Departamento de Clínica Médica, Goiânia, GO, Brasil; 6 Universidade Evangélica de Goiás, Faculdade de Medicina, Anápolis, GO, Brasil; 7 Hospital de Campanha para o Enfrentamento ao Coronavírus de Goiânia, Goiânia, GO, Brasil; 8 Hospital Estadual de Doenças Tropicais Dr. Anuar Auad, Goiânia, GO, Brasil; 9 Universidade Federal de Goiás, Faculdade de Medicina, Departamento de Pediatria, Goiânia, GO, Brasil

**Keywords:** Covid-19, Hospitalização, Mortalidade, Estudos de Coorte, Brasil

## Abstract

**Objetivo:**

Descrever aspectos clínico-epidemiológicos e terapêuticos e estimar letalidade e fatores de risco para menor sobrevida intra-hospitalar por covid-19.

**Métodos:**

Trata-se de estudo de coorte retrospectiva conduzido em Goiás, em 2020, com dados obtidos a partir do Sistema de Informação de Vigilância Epidemiológica da Gripe e por meio de revisão de prontuários clínicos e registros hospitalares. Foi estimado risco relativo para o óbito intra-hospitalar e realizadas análises de regressão múltipla de Poisson e regressão de Cox. As funções de sobrevida foram comparadas pelo teste log-rank e representadas pelas curvas de Kaplan-Meier.

**Resultados:**

A amostra foi composta por 651 adultos, cuja mediana de idade era 59 anos, 57,0% internados em hospital público, 61,1% com síndrome respiratória aguda grave à admissão e 72,0% com alguma comorbidade, sendo as mais frequentes hipertensão arterial, diabetes e obesidade. A letalidade global foi 17,5% (intervalo de confiança 95%, IC95% 14,7; 20,6), sem diferença significativa entre hospital público e privado. A letalidade foi maior na faixa etária ≥60 anos (razão de prevalência, RP 1,26; IC95% 1,01; 1,58), em hipertensos (RP 1,41; IC95% 1,14; 1,74) e naqueles sob cuidados intensivos (RP 2,68; IC95% 1,13; 6,32) e ventilação mecânica (RP 11,15; IC95% 5,53; 22,46). A mediana de tempo entre a admissão hospitalar e o óbito foi de 10 dias (intervalo interquartílico, 6-18). A sobrevida foi menor no grupo ≥60 anos (*hazard ratio* ajustada, HR 1,93; IC95% 1,26; 2,95) e naquele sob ventilação mecânica (HR 10,13; IC95% 6,03; 17,02).

**Conclusão:**

Fatores relacionados a comorbidades e gravidade foram preditores independentes de menor sobrevida intra-hospitalar em pacientes com covid-19.

**Aspectos éticos**

A presente pesquisa respeitou os princípios éticos, obtendo os seguintes dados de aprovação:

**Comitês de ética em pesquisa**: **Números dos pareceres**, **Datas de aprovação**

Hospital das Clínicas da Universidade Federal de Goiás: 4515457 5112519, 29/1/2021 18/11/2021

Hospital e Maternidade Dona Íris: 4869484 5301886, 27/7/2021 21/3/2022

Leide das Neves Ferreira (Secretaria Estadual de Saúde): 5171067, 16/12/2021

Registro de consentimento livre e esclarecido: Dispensado pelos comitês

## Introdução

A covid-19 é uma doença sistêmica, multiforme e grave, com sintomas e desfechos variáveis, diferindo de acordo com a população afetada, os cenários epidemiológicos, as variantes circulantes e o status da vacinação (1). A pandemia de covid-19 impôs desafios significativos aos sistemas de saúde em todo o mundo. O Brasil, um dos países mais afetados, enfrentou questões únicas devido à sua grande população, à extensão e às disparidades regionais na infraestrutura de saúde e vulnerabilidade socioeconômica (1,2). No Brasil, o número de casos atingiu mais de 37 milhões e cerca de 703 mil mortes até junho de 2023 (3). 

A letalidade por covid-19 no Brasil foi estimada em 35% a 38% para os pacientes hospitalizados, chegando a 59% naqueles internados em unidades de terapia intensiva e até 80% naqueles ventilados mecanicamente (4,5). Observam-se diferenças regionais nas taxas de letalidade e no perfil clínico de indivíduos internados em diferentes fases da pandemia (2,4-6). Embora exista vasta literatura utilizando dados nacionais ou regionais, até o momento, poucos estudos analisaram detalhadamente os aspectos clínicos e o manejo terapêutico de pacientes internados em hospital público e privado no Centro-Oeste brasileiro (7).

Este estudo objetivou descrever aspectos clínico-epidemiológicos e manejo terapêutico, além de estimar a letalidade e os fatores de risco para óbito e menor sobrevida intra-hospitalar em adultos hospitalizados por covid-19 em Goiás, Brasil, em 2020. 

## Métodos

### Tipo de estudo

Trata-se de estudo de coorte retrospectiva. 

### Contexto 

O estudo foi realizado em três hospitais públicos e dois hospitais privados em Goiânia e Anápolis, em Goiás, Brasil. Esses hospitais integravam a rede de assistência especializada a pacientes com covid-19. Em 2020, 34 unidades de saúde dispunham de leitos em unidades de terapia intensiva para adultos com covid-19, das quais 27 eram em Goiânia; 7, em Anápolis; 20, públicas; e 14, privadas. Goiás tem a população estimada em 7 milhões de habitantes, sendo Goiânia a capital (1.437.366 habitantes) e Anápolis a maior cidade fora da região metropolitana (398.869 habitantes) (8). 

### Participantes

Foram incluídos indivíduos com idade ≥18 anos, hospitalizados entre março e dezembro de 2020 por covid-19, confirmada por teste molecular ou teste antigênico. Pacientes que permaneceram hospitalizados por menos de 24 horas ou transferidos foram excluídos da amostra.

### Variáveis

O desfecho primário foi a morte intra-hospitalar. Os desfechos secundários foram: internação em unidades de terapia intensiva, necessidade de ventilação mecânica, drogas vasoativas, hemodiálise, tempo de internação até o óbito ou alta do paciente, tempo de ventilação, tempo em cuidados intensivos e taxas de letalidade por subgrupo.

As variáveis de exposição foram agrupadas conforme a seguir.

a) Dados sociodemográficos: sexo (feminino, masculino); faixa etária (em anos: <30, 30-59, ≥60); raça/cor da pele (branca, parda/preta, outras); hospital (público, privado); e período de hospitalização (março-maio, junho-agosto, setembro-dezembro).
b) Condições subjacentes (categorizadas de forma dicotômica – sim, não): comorbidade; obesidade; hipertensão arterial sistêmica; diabetes; doença pulmonar obstrutiva crônica; doença renal crônica; e gravidez.
c) Sintomas (categorizados de forma dicotômica – sim, não): febre; dispneia; e síndrome respiratória aguda grave à admissão hospitalar.
d) Exame de imagem do tórax (radiografia e tomografia computadorizada): comprometimento pulmonar (<50%, ≥50%), considerando-se os exames realizados.
e) Terapêuticas (categorizadas de forma dicotômica – sim, não): internação em cuidados intensivos; necessidade de ventilação mecânica; uso de antibióticos; uso de corticosteroides; uso de droga vasoativa; uso de heparina; e necessidade de hemodiálise. O uso de dose plena de heparina (1mg/kg, de 12/12 horas) foi comparado à dose profilática de heparina (1mg/kg/dia), dentre os que utilizaram heparina.
f) Exames complementares (valores com maior alteração ao longo da internação): anemia (hemoglobina <12g/L, ≥12g/L); leucocitose (leucócitos>10.000 células/µL, 4.000-10.000 células/µL); linfopenia (linfócitos <1.000 células/µL, ≥1.000 células/µL); plaquetopenia (plaquetas <100.000 células/µL, ≥100.000 células/µL); alterações de enzimas hepáticas (alanina aminotransferase e aspartato aminotransferase >40 U/L, ≤40 U/L); desidrogenase lática (>328 U/L, ≤328 U/L); injúria renal (creatinina ≥1,4mg/dL, <1,4mg/dL); e dímero D (≥500 µg/L, <500 µg/L).

### Fonte dos dados

Os dados foram obtidos a partir do Sistema de Informação de Vigilância Epidemiológica da Gripe e por meio de revisão de prontuários clínicos e registros hospitalares. Foram avaliadas as características clínicas, os exames complementares e o manejo terapêutico, desde a admissão até a alta ou o óbito hospitalar. Os casos foram categorizados de acordo com a classificação clínica da Organização Mundial da Saúde (9) em leves, moderados, graves e críticos. 

Os dados do estudo foram coletados e gerenciados usando a plataforma de captura eletrônica de dados (Research Electronic Data Capture – REDCap), hospedada no servidor da Universidade Federal de Goiás. O formulário de dados foi elaborado pelos pesquisadores, e a coleta foi realizada por profissionais treinados da área da saúde. 

### Tamanho do estudo

Realizou-se a amostragem não probabilística de pacientes internados em cada um dos hospitais participantes. Contabilizou-se que a amostra de 650 pessoas seria suficiente para estimar um risco relativo (RR) de 1,80 para o desfecho primário (óbito durante internação hospitalar), tendo como exposição a presença de pelo menos uma comorbidade. Os parâmetros utilizados foram nível de significância bicaudal (1-alfa) de 95%; poder de 80% (1-beta); razão entre expostos e não expostos igual a 2; e 15% dos não expostos e 24% dos expostos apresentarem o desfecho primário.

### Métodos estatísticos

Os dados foram analisados utilizando o programa IBM SPSS 20.0. Calcularam-se mediana e intervalo interquartílico (IIQ) para as variáveis contínuas e descritas frequências absolutas (n) e relativas (%) para as variáveis categóricas. A taxa de letalidade foi calculada pelo número de indivíduos hospitalizados que evoluíram para óbito durante a hospitalização nos diferentes subgrupos. Para identificar fatores associados ao óbito, inicialmente, foram calculados os RR, com seus respectivos intervalos de confiança de 95% (IC95%), para cada variável. Os dados de RR referentes aos exames laboratoriais foram apresentados separadamente.

O modelo de regressão múltipla de Poisson com estimador de variância robusta foi realizado para analisar os fatores preditivos de letalidade, apresentados com suas razões de prevalência (RP) e IC95%; p-valor<0,05 foi considerado estatisticamente significativo para todos os testes. As variáveis colineares ou com mais de 10% de valores faltantes (exames laboratoriais, imagem do tórax e heparina) não foram incluídas em análises múltiplas, assim como aquelas que apresentavam p-valor≥0,10 na análise bivariada.

Os grupos tiveram suas funções de sobrevivência comparadas pelo teste log-rank e representadas graficamente pelas curvas de Kaplan-Meier para as variáveis faixa etária (em anos: <60, ≥60), hipertensão arterial, injúria renal (creatinina ≥1,^4^), ventilação mecânica, cuidados intensivos e gravidez. Essa análise foi complementada pelo modelo de regressão de Cox bivariada, obtendo-se a razão de risco bruta (*hazard ratio*, HR). As variáveis com p-valor≤0,10 na análise bivariada foram incluídas no modelo de riscos proporcionais de Cox para ajustar possíveis fatores de confusão. A análise de riscos proporcionais de Cox foi realizada em modelo retroativo (backwards), incluindo as variáveis sexo, idade, comorbidades, síndrome respiratória aguda grave à admissão, necessidade de cuidados intensivos e ventilação mecânica. A magnitude do efeito final foi obtida apresentando-se a HR e o IC95%. O nível de significância foi obtido com o teste de Wald (p-valor<0,05). O teste de Schoenfeld foi utilizado para análise de resíduos.

## Resultados

Foram incluídos 651 adultos com covid-19: 50,5% eram do sexo feminino, 63,6% foram internados de junho a agosto de 2020 e 57,0% foram internados em hospital público. A mediana de idade foi de 59 anos (IIQ 44-71 anos). Dos 651 adultos com covid-19, 61,1% foram admitidos como síndrome respiratória aguda grave, 56,0% foram classificados como graves ou críticos segundo a Organização Mundial de Saúde e 72,0% tinham alguma comorbidade, sendo as mais frequentes hipertensão, diabetes e obesidade (Tabela 1). 

**Tabela 1:** Letalidade (%) por subgrupo, risco relativo (RR) e intervalos de confiança de 95% (IC95%) para características sociodemográficas e condições clínicas da população do estudo. Goiás, 2020 (n=651)

Características	Total n (%)	Letalidade n (%)	RR (IC95%)	p-valor^a^
Sexo				
Feminino	329 (50,5)	53 (18,9)	1,00	0,342
Masculino	322 (49,5)	61 (16,1)	1,03 (0,96; 1,11)	
**Faixa etária** (anos)				
<30	47 (7,2)	2 (4,3)	1,00	<0,001
30-59	291 (44,7)	29 (10,0)	1,06 (0,99; 1,14)	
≥60	313 (48,1)	83 (26,5)	1,30 (1,19; 1,42)	
**Raça/cor da pele**				
Branca	104 (37,0)	17 (16,3)	1,00	
Parda/preta	162 (57,7)	27 (16,6)	0,99 (0,89; 1,11)	0,945
Outras	15 (5,3)	-		
Hospital				
Privado	280 (43,0)	48 (17,1)	0,96 (0,68; 1,35)	0,830
Público	371 (57,0)	66 (17,8)	1,00	
**Período de hospitalização**				
Março-maio	104 (16,0)	22 (21,2)	1,12 (0,99; 1,25)	
Junho-agosto	414 (63,6)	76 (18,4)	1,08 (0,99; 1,16)	0,058
Setembro-dezembro	133 (20,4)	16 (12,0)	1,00	
**Condições subjacentes**				
Comorbidade (no mínimo uma)	469 (72,0)	100 (21,3)	1,17 (1,10; 1,25)	<0,001
Comorbidade ausente	182 (28,0)	14 (7,7)	1,00	
Obesidade presente	163 (25,0)	30 (26,3)	1,01 (0,93; 1,10)	0,729
Obesidade ausente	488 (75,0)	84 17,2)	1,00	
Hipertensão presente	319 (49,0)	79 (24,8)	1,19 (1,10; 1,28)	<0,001
Hipertensão ausente	332 (51,0)	35 (10,5)	1,00	
Diabetes presente	184 (28,3)	53 (28,8)	1,22 (1,11; 1,35)	<0,001
Diabetes ausente	467 (71,7)	61 (13,0)	1,00	
Doença pulmonar obstrutiva crônica presente	35 (5,4)	11 (31,4)	1,21 (0,97; 1,52)	0,030
Doença pulmonar obstrutiva crônica ausente	616 (94,6)	103 (16,7)	1,00	
Doença renal crônica (prévia) presente	31 (4,8)	11 (35,5)	1,29 (0,99; 1,68)	0,007
Doença renal crônica (prévia) auesente	620 (95,2)	103 (16,6)	1,00	
Gravidez presente	36 (10,9)	4 (11,1)	0,94 (0,83; 1,06)	0,387
Gravidez ausente	293 (89,1)	49 (16,7)	1,00	
Sintomas				
Febre presente	244 (37,5)	61 (25,0)	1,16 (1,07; 1,26)	<0,001
Febre ausente	407 (62,5)	53 (13,0)	1,00	
Dispneia presente	539 (82,8)	107 (19,9)	1,17 (1,09; 1,25)	0,001
Dispneia ausente	112 (17,2)	7 (6,3)	1,00	
Síndrome respiratória aguda grave à admissão presente	398 (61,1)	91 (22,9)	1,18 (1,10; 1,16)	<0,001
Síndrome respiratória aguda grave à admissão ausente	253 (38,9)	23 (9,1)	1,00	
**Exame de imagem do tórax**				
Envolvimento pulmonar ≥50%	197 (33,9)	77 (39,1)	1,54 (1,37; 1,73)	<0,001
Envolvimento pulmonar <50%	384 (66,1)	24 (6,2)	1,00	
**Medidas terapêuticas**				
Cuidados intensivos presentes	207 (31,8)	103 (49,8)	1,94 (1,69; 2,22)	<0,001
Cuidados intensivos ausentes	444 (68,2)	11 (24,7)	1,00	
Ventilação mecânica invasiva presente	107 (16,4)	96 (89,7)	9,40 (5,37; 16,46)	<0,001
Ventilação mecânica invasiva ausente	544 (83,6)	18 (3,3)	1,00	
Antibiótico presente	579 (88,9)	110 (19,0)	1,17 (1,09; 1,25)	0,008
Antibiótico ausente	72 (11,1)	4 (5,5)	1,00	
Corticosteroide presente	445 (68,4)	87 (19,6)	1,08 (1,01; 1,16)	0,044
Corticosteroide ausente	206 (31,6)	27 (13,1)	1,00	
Droga vasoativa presente	96 (14,7)	76 (79,2)	4,47 (3,03; 6,61)	<0,001
Droga vasoativa ausente	555 (85,3)	38 (6,8)	1,00	
Heparina presente	539 (82,8)	97 (18,0)	1,03 (0,95; 1,13)	0,475
Heparina ausente	112 (17,2)	17 (15,0)	1,00	
Dose plena de heparina (1mg/kg de 12/12 horas)	61 (11,3)	23 (37,7)	1,36 (1,11; 1,65)	<0,001
Dose profilática de heparina (1mg/kg/dia)	478 (88,6)	74 (15,4)	1,00	
Hemodiálise presente	54 (8,3)	42 (77,8)	3,96 (2,40; 6,52)	<0,001
Hemodiálise ausente	597 (91,7)	72 (12,0)	1,00	

^a^Teste Qui-quadrado (p<0.05).

A letalidade foi de 17,5% (IC95% 14,7; 20,^6^), sem diferenças estatisticamente significativas entre a rede pública e a rede privada (p-valor>0,05). Houve maior proporção de casos classificados como graves ou críticos nos hospitais públicos (69,0% vs. 40,0%, p-valor<0,001). Entre os pacientes que foram a óbito, 88,0% tinham alguma comorbidade e a letalidade foi mais elevada nesses se comparada àqueles sem comorbidades (21,3% vs. 7,7%; p-valor<0,001). A letalidade entre os pacientes em cuidados intensivos foi de 49,8% (RR 1,94, IC95% 1,69; 2,^22^) e naqueles em ventilação mecânica foi de 89,7% (RR 9,40, IC95% 5,37; 16,46) (Tabela 1).

As gestantes representaram 5,5% (n=36), sendo 35 delas internadas em hospital público. A mediana de idade foi 24 anos (IIQ 24-) e 31 semanas de idade gestacional (IIQ 26-); todas apresentavam pelo menos uma comorbidade. A admissão em cuidados intensivos ocorreu em 36,1% delas, e a ventilação mecânica, em 22,2%. A taxa de letalidade foi de 11,1% (IC95% 3,10; 26,^10^).

Hemoglobina <12g/L; leucócitos >10.000 células/µL; plaquetas <100.00/µL; bem como valores de lactato desidrogenase, aminotransferases, creatinina e dímero-D acima dos valores de referência, mostraram associação com maior risco de óbito em análise bivariada (Tabela 2). 

**Tabela 2:** Letalidade (%), risco relativo (RR) e intervalos de confiança de 95% (IC95%) para óbito intra-hospitalar, por variáveis laboratoriais registradas durante a internação. Goiás, 2020

Exames laboratoriais	Total n (%)	Óbitos n (%)	RR (IC95%)	p-valor^a^
Hemoglobina <12g/L	180 (51,9)	60 (33,3)	1,40 (1,25; 1,56)	<0,001
Hemoglobina ≥12g/L	167 (48,1)	11 (6,5)	1,00	
Leucócitos >10.000 células/µL	185 (61,5)	69 (37,3)	1,54 (1,37; 1,73)	<0,001
Leucócitos ≤10.000 células/µL	116 (38,5)	4 (3,4)	1,00	
Linfócitos <1.000 células/µL	180 (52,6)	32 (17,8)	0,91 (0,81; 1,01)	0,090
Linfócitos ≥1.000 células/µL	162 (47,4)	41 (25,3)	1,00	
Plaquetas <100.000/µL	56 (16,1)	22 (39,2)	1,40 (1,13; 1,74)	<0,001
Plaquetas ≤100.000/µL	291 (83,9)	43 (14,7)	1,00	
Lactato desidrogenase >328 U/L	167 (75,2)	48 (28,7)	1,30 (1,15;1,47)	0,001
Lactato desidrongenase ≤328 U/L	55 (24,8)	4 (7,2)	1,00	
Aminotransferase, alanina >40 U/L	158 (63,2)	42 (26,6)	1,19 (1,06; 1,35)	0,008
Aminotransferase, alanina ≤40 U/L	92 (36,8)	11 (11,9)	1,00	
Aminotransferase, aspartato >40 U/L	174 (70,4)	50 (28,7)	1,35 (1,21; 1,49)	<0,001
Aminotransferase, aspartato ≤40 U/L	73 (29,6)	3 (4,1)	1,00	
Creatinina ≥1,4mg/dL (injúria renal)	115 (36,3)	71 (61,7)	2,56 (2,03; 3,23)	<0,001
Creatinina <1,4mg/dL	202 (63,7)	4 (19,8)	1,00	
Dímero D≥500 µg/L	182 (67,2)	57 (31,3)	1,39 (1,25; 1,55)	<0,001
Dímero D<500 µg/L	89 (32,8)	4 (4,5)	1,00	

^a^Teste Qui-quadrado (p<0.05).

Na análise de regressão de Poisson com estimador robusto, as variáveis demográficas e clínicas que se mostram independentemente associadas ao óbito foram idade ≥60 anos (RP 1,26; IC95% 1,01; 1,58), hipertensão arterial (RP 1,14; IC95% 1,14; 1,74), internação em cuidados intensivos (RP 2,68; IC95% 1,13; 6,32) e necessidade de ventilação mecânica (RP 11,15; IC95% 5,53; 22,46) (Tabela 3). Os resultados laboratoriais preditores de óbito foram a contagem de leucócitos >10,000 células/µL (RP 1,16; IC95% 1,02; 1,32) e creatinina ≥1,4mg/dL (RP 1,65; IC95% 1,31; 2,08) (Tabela suplementar 1). 

**Tabela 3:** Razões de prevalência (RP) e intervalos de confiança de 95% (IC95%) ajustadas para o óbito intrahospitalar por covid-19 após regressão de Poisson com estimador de variância robusta. Goiás, 2020 (n=651)

Características	RP (IC95%)	p-valor^a^
Sexo masculino	0,96 (0,80; 1,16)	0,696
Sexo feminino	1,00	
Idade ≥60 anos	1,26 (1,01; 1,58)	0,039
Idade <60 anos	1,00	
Hipertensão arterial presente	1,41 (1,14; 1,74)	<0,001
Hipertensão arterial ausente	1,00	
Cuidados intensivos presentes	2,68 (1,13; 6,32)	0,025
Cuidados intensivos ausentes	1,00	
Ventilação mecânica invasiva presente	11,15 (5,53; 22,46)	<0,001
Ventilação mecânica invasiva ausente	1,00	

^a^Teste de Wald (p<0.05).

A mediana de tempo entre a admissão hospitalar e o óbito foi de 10 dias (IIQ 6-18) – 70,0% ocorreram em até 14 dias. Entre os admitidos em unidades de terapia intensiva, a mediana de permanência hospitalar foi de 9 dias (IIQ 6-14 dias), sendo 6 dias (IIQ 3-11 dias) em cuidados intensivos e 7 dias (IIQ 4-13 dias) em ventilação mecânica. O tempo de hospitalização foi maior entre os não sobreviventes quando comparado aos que tiveram alta hospitalar (10 vs. 6 dias). Aqueles que foram a óbito receberam mais antibióticos (96,5% vs. 87,3%) e corticosteroides (76,3% vs. 66,7%) e usaram heparina em dose plena (23,7% vs. 8,6%). 

Faixa etária, hipertensão arterial, injúria renal, gravidez, necessidade de ventilação mecânica e de cuidados intensivos mostraram associação com o óbito em análise de regressão de Cox bivariada (p-valor<0,05) (Figura 1). O p-valor e a representação gráfica de cada variável obtidos após teste de Schoenfeld estão disponíveis em material suplementar (Tabela suplementar 2 e Figura suplementar 1). Não houve violação do pressuposto de proporcionalidade para as covariáveis utilizadas, exceto sexo. Após a análise de riscos proporcionais de Cox, apenas as variáveis idade ≥60 anos (HR 1,93; IC95% 1,26; 2,95) e uso de ventilação mecânica (HR 10,13; IC95% 6,03; 17,02) foram independentemente associadas a uma menor sobrevida intra-hospitalar (Figura 2), mesmo quando avaliados apenas pacientes que necessitaram cuidados intensivos. 

**Figura 1:** Tempo de sobrevivência (dias de hospitalização) por variáveis preditoras de óbito – idade (A), hipertensão arterial (B), injúria renal (C), ventilação mecânica (D), cuidados intensivos (E) e gravidez (F) – durante o período de internação hospitalar. Goiás, 2020

**Figura 2:** Sobrevida de acordo com idade (A) e ventilação mecânica (B). Goiás, 2020 (n=651)

## Discussão

Neste estudo, analisaram-se os aspectos clínicos, laboratoriais, terapêuticos e de fatores associados ao óbito de adultos hospitalizados por covid-19 em Goiás no primeiro ano da pandemia. Os pacientes que morreram eram mais velhos, tinham mais comorbidades e apresentavam maior gravidade clínica à admissão e necessidade de cuidados intensivos. A idade ≥60 anos, a hipertensão arterial e o uso de ventilação invasiva foram fatores independentemente associados à maior letalidade. Idade e presença de comorbidades relacionam-se a maior necessidade de cuidados avançados de vida e com os desfechos desfavoráveis, incluindo óbito, em pessoas com covid-19 (10,11). O adequado controle de fatores potencialmente modificáveis, tais como diabetes, hipertensão arterial, obesidade, são fundamentais para a redução de letalidade por síndromes respiratórias graves. 

Evidenciaram-se similaridades entre as características desta amostra com estudos conduzidos no Sul e no Sudeste brasileiro em relação à frequência de comorbidades (79,8%) e de internações em hospital público (52,8%) (12,13). Nessas regiões, a letalidade variou de 19,0% a 34,4% (5,12,13). No Norte e no Nordeste, a letalidade hospitalar foi de 41,0% a 44,0%, chegando a 96,0% naqueles sob ventilação mecânica em 2020 (5,14). Houve letalidade de 21,0% (IC95% 18,0; 24,0) em hospitais de várias regiões do Brasil, variando de 9,0% e 48,0%, com associação positiva entre maior sobrevida e melhores indicadores econômicos da região onde os pacientes foram atendidos (15).

Uma coorte multicêntrica conduzida em 25 centros brasileiros, em 2020, encontrou preditores para letalidade intra-hospitalar similares aos resultados desta pesquisa, tais como idade ≥65 anos, hipertensão arterial, ventilação invasiva e plaquetas <100.000 células/L (13). Neste estudo, alterações laboratoriais como leucocitose, anemia, plaquetopenia e elevação de transaminases, desidrogenase lática, dímero D e creatinina representaram risco para óbito (p-valor<0,05).

Não foram identificadas diferenças estatisticamente significativas na letalidade entre hospital público e privado, apesar das apresentações graves e críticas serem mais frequentes na rede pública de saúde. Coortes brasileiras evidenciaram menor mortalidade entre os internados em hospital privado (13,16,17), porém hospital público recebeu maior proporção de pacientes de alto risco ou com maior gravidade (15).

Durante a primeira onda da pandemia de covid-19, a taxa de letalidade reportada em diferentes regiões do Brasil foi de 6,5% a 10,8% em hospital privado (13,16,17) e, na rede pública, de 21,7%, variando de 31,8% na primeira a 18,2% na segunda onda (18). Isso poderia ser explicado pelas disparidades regionais no que tange a desigualdades sociais e de acesso à saúde, somado a períodos de escassez de insumos, recursos humanos e leitos hospitalares enfrentados pelo sistema de saúde nas diferentes fases da pandemia (13,15,18).

Na pandemia de covid-19, as diretrizes de manejo clínico foram estabelecidas à medida que se dispunha de evidências. Neste estudo, 68,4% dos pacientes utilizaram corticoides, enquanto diretrizes atuais preconizam o uso apenas em casos em que há necessidade de oxigênio (9). Apesar da baixa ocorrência de coinfecções bacterianas e fúngicas efetivamente documentadas (19,20), 88,9% dos pacientes utilizaram antibióticos, ainda maior do que os 74,6% (IC95% 68,3; 80,0) reportados em uma metanálise (21). O uso excessivo de agentes antimicrobianos durante a pandemia já impactou negativamente as taxas de resistência antimicrobiana e é considerado uma herança preocupante (22,23).

Embora a gravidez não tenha sido identificada como fator de risco para o óbito em análises multivariadas, a letalidade de 11,0% foi preocupante. Até junho de 2021, a taxa geral de letalidade por covid-19 entre mulheres grávidas e no pós-parto no Brasil era de 7,2% (24). Todas as gestantes desta coorte seriam consideradas como alto risco por possuírem ao menos uma comorbidade. Vale lembrar que dois dos locais do estudo eram referência para gestantes com covid-19, o que poderia tê-los tornado escolhas prioritárias para casos graves e, assim, impactado na letalidade nessa população. 

Este estudo apresentou limitações inerentes aos estudos retrospectivos, considerando a qualidade e a finalidade dos registros disponíveis. A utilização de amostra de conveniência e não probabilística pode não representar a população de pacientes hospitalizados. Vários fatores de risco foram avaliados de forma dicotômica, sem estratificação por gravidade. Soma-se a isso as diferenças de manejo entre hospitais e a incompletude de dados registrados em prontuários clínicos. Para garantir a uniformização, utilizou-se formulário padronizado para coleta de dados, e a revisão de prontuários foi realizada por profissionais de saúde adequadamente treinados.

Este estudo descreve dados de vários hospitais de Goiás, com informações clínico-epidemiológicas, laboratoriais e terapêuticas detalhadas, incluindo a análise de sobrevida. Concluiu-se que fatores relacionados a comorbidades, como idade avançada e hipertensão arterial, assim como aqueles associados a uma maior gravidade clínica, foram preditores de óbito e de menor sobrevida intra-hospitalar. Compreender os fatores de risco para a morte e as disparidades regionais enfrentadas durante o início da pandemia de covid-19 no Brasil é fundamental para auxiliar melhorias na gestão da saúde e fornecer subsídios para reduzir morbimortalidade durante o surgimento de epidemias de síndromes respiratórias agudas graves.

## Data Availability

O banco de dados utilizado na pesquisa está disponível em: https://figshare.com/s/6337256d2548a3f0ed5f.
